# EEG resting state alpha dynamics predict an individual’s vulnerability to auditory hallucinations

**DOI:** 10.1007/s11571-024-10093-1

**Published:** 2024-03-22

**Authors:** H. Honcamp, S. X. Duggirala, J. Rodiño Climent, A. Astudillo, N. J. Trujillo-Barreto, M. Schwartze, D. E. J. Linden, T. A. M. J. van Amelsvoort, W. El-Deredy, S. A. Kotz

**Affiliations:** 1 Department of Neuropsychology and Psychopharmacology, Faculty of Psychology and Neuroscience, Maastricht University, Universiteitssingel 40, 6229 ER Maastricht, The Netherlands; 2 Brain Dynamics Laboratory, Universidad de Valparaíso, Valparaiso, Chile; 3 NICM Health Research Institute, Western Sydney University, Penrith, NSW Australia; 4 School of Health Sciences, University of Manchester, Manchester, UK; 5 Department of Psychiatry and Neuropsychology, School of Mental Health and Neuroscience, Maastricht University Medical Center, Maastricht, The Netherlands; 6 Centro de Investigación y Desarrollo en Ingeniería en Salud, Universidad de Valparaíso, Valparaiso, Chile

**Keywords:** Alpha, EEG resting state, Hallucination proneness, Hidden semi-Markov modeling, Risk assessment, Temporal dynamics

## Abstract

Task-free brain activity exhibits spontaneous fluctuations between functional states, characterized by synchronized activation patterns in distributed resting-state (RS) brain networks. The temporal dynamics of the networks’ electrophysiological signatures reflect individual variations in brain activity and connectivity linked to mental states and cognitive functions and can predict or monitor vulnerability to develop psychiatric or neurological disorders. In particular, RS alpha fluctuations modulate perceptual sensitivity, attentional shifts, and cognitive control, and could therefore reflect a neural correlate of increased vulnerability to sensory distortions, including the proneness to hallucinatory experiences. We recorded 5 min of RS EEG from 33 non-clinical individuals varying in hallucination proneness (HP) to investigate links between task-free alpha dynamics and vulnerability to hallucinations. To this end, we used a dynamic brain state allocation method to identify five recurrent alpha states together with their spatiotemporal dynamics and most active brain areas through source reconstruction. The dynamical features of a state marked by activation in somatosensory, auditory, and posterior default-mode network areas predicted auditory and auditory-verbal HP, but not general HP, such that individuals with higher vulnerability to auditory hallucinations spent more time in this state. The temporal dynamics of spontaneous alpha activity might reflect individual differences in attention to internally generated sensory events and altered auditory perceptual sensitivity. Altered RS alpha dynamics could therefore instantiate a neural marker of increased vulnerability to auditory hallucinations.

**Supplementary Information:**

The online version contains supplementary material available at 10.1007/s11571-024-10093-1.

## Introduction

In the absence of a cognitive task, the brain spontaneously fluctuates between large-scale functional networks—the resting state networks (RSNs). The temporal dynamics of the resting state (RS) can be characterized by a sequence of recurring brain states with unique functional connectivity (FC) and spatiotemporal characteristics (Baker et al. 2014; Hutchison et al. 2013; Trujillo-Barreto et al. 2024; Vidaurre et al. 2016; Woolrich et al. 2013). The dynamical features of the states, such as their dwell time and transitioning behavior relate to cognition and behavior in neurotypical and patient populations and could therefore inform about early vulnerability to psychopathology (Damoiseaux et al. 2006; Kottaram et al. 2019; Nishida et al. 2013; Vidaurre et al. 2016). However, these states cannot be directly observed in functional imaging or electrophysiological recordings and must be inferred. State allocation methods have been developed to identify the brain states and their dynamical features and relate them to task performance and cognitive and mental states (Baker et al. 2014; Trujillo-Barreto et al. 2024).

Auditory verbal hallucinations (AVH), the untriggered experience of voices in the absence of external stimulation, display in neuropsychiatric disorders with psychotic features as well as in the general population (Bartels-Velthuis et al. 2010; Linszen et al. 2022). Thus, AVH are not necessarily related to the need for care but lie on a continuum of vulnerability, called hallucination proneness (HP) (Johns et al. 2014; Johns and Van Os 2001). The continuum perspective suggests that the underlying cognitive mechanisms and neuronal changes associated with AVH in neurotypical individuals are an attenuated version of those seen in clinically diagnosed individuals (Allen et al. 2005; Badcock and Hugdahl 2012; Diederen et al. 2012; Marschall et al. 2023; Paulik et al. 2008; Pinheiro et al. 2019). Interestingly, auditory, but not visual, perceptual aberrancies predict conversion to psychosis, suggesting distinct neurocognitive substrates underlying hallucinatory experiences across different modalities (Lehembre-Shiah et al. 2017).

Currently, little is known about the underlying neurophysiology of the non-clinical (auditory) HP continuum where marked symptoms are absent, but individuals may be at risk of developing them. Considering the fluctuating and unprovoked nature of hallucinatory experiences, the temporal dynamics of the resting brain might be a suitable target to elucidate their neural basis (Alderson-Day et al. 2015). The ‘resting state hypothesis of AVH’ suggests that AVH result from a dysfunctional interaction, i.e., aberrant bottom-up and top-down processes between the Default-Mode Network (DMN) and other RSNs, including the salience, cognitive control, and auditory networks (Northoff and Qin 2011). The DMN is known to drive spontaneous, internally directed thoughts and perceptions (Damoiseaux et al. 2006; Raichle 2015). Thus, altered temporal dynamics of the DMN and other networks could result in an increased focus on, and altered sensory sensitivity to internally generated cues (e.g., spontaneously retrieved memories or intrusive inner speech). If additionally combined with altered auditory processing, this could underlie self-monitoring and source attribution difficulties in hallucination-prone individuals and those with AVH (Brookwell et al. 2013; Horga et al. 2014; van Lutterveld et al. 2014). Moreover, it was reported that disturbed sensory responsiveness to internally generated stimuli is related to the presence of auditory hallucinations rather than to the diagnosis of a psychotic disorder (Blakemore et al. 2000; Lewis-Hanna et al. 2011).

Electroencephalography (EEG) alpha band activity (8–12 Hz) is associated with cortical excitability and selective attention across sensory domains (Hindriks et al. 2017). Alpha power fluctuations modulate the sensitivity to sensory experiences, altering the perception threshold for visual, tactile, and pain stimuli (Craddock et al. 2017; Ecsy et al. 2017; Klimesch et al. 2007). Alpha activity further influences the temporal and spatial grouping of sensory information when perceiving ambiguous stimuli, indicating that alpha activity shapes the way sensory information is attended to and perceived (Shen et al. 2019). Alpha fluctuations are also associated with cognitive control (Clements et al. 2021), and deficits in cognitive control such as intrusive thoughts and impaired reality-checking are related to HP, psychosis proneness, and schizotypy (Alderson-Day et al. 2019; Paulik et al. 2008; Waters et al. 2012). Although not exclusively mapped, alpha activity has been linked to the DMN (Hillebrand et al. 2012; Samogin et al. 2020). Hence, aberrant alpha dynamics may inform about individual variability in sensory responsiveness underlying the self-monitoring difficulties related to AVH in clinical and non-clinical individuals and HP (Blakemore et al. 2000; Lewis-Hanna et al. 2011).

Given the time-varying nature of RS brain activity, the importance of alpha activity in attention and sensory responsiveness, and its link to DMN activation, analyzing RS alpha dynamics can uncover neural correlates along the HP continuum. To accurately characterize these dynamics on a sub-second scale, we used the Hidden semi-Markov Model (HsMM), a Brain State allocation method that identifies quasi-stable activity patterns with distinct temporal properties (Baker et al. 2014). The HsMM thereby reveals relevant information about network connectivity and switching dynamics (Baker et al. 2014; Hunyadi et al. 2019; Trujillo-Barreto et al. 2024; Woolrich et al. 2013). The HsMM is a generalization of the classic Hidden Markov Model (HMM) and allows incorporating more realistic assumptions about long-range dependencies of M/EEG time series and network switching behavior by explicit modeling of the state durations (Trujillo-Barreto et al. 2024). Thus, it provides a means to accurately assess the electrophysiological spatiotemporal characteristics underlying spontaneous, dynamic, and transient phenomena, such as hallucinatory experiences. The increased temporal sensitivity of the HsMM facilitates detecting subtle alterations in RS dynamics in non-clinical hallucination-prone individuals that might have been missed by alternative, less temporally sensitive methods (Honcamp et al. 2022). Hence, the application of the HsMM might inform about early vulnerability and corresponding neurophysiological HP correlates.

Here, we applied the HsMM to the temporally concatenated RS EEG alpha envelope of non-clinical individuals to analyze the predictive value of state durations and occupancy for general, auditory, and auditory-verbal HP. Additionally, we identified the neural sources that correspond to each state through frequency-domain source localization to confirm correspondence with RSNs implicated in hallucinatory predisposition.

## Methods and materials

### Participants and procedure

The current study is part of a research line into brain mechanisms of hallucinations and hallucination proneness at Maastricht University, the Netherlands. Ethical approval was granted by the medical ethics committee responsible (METC-20 035; the study was ended early due to recruitment difficulties). Inclusion criteria for participation were age between 16 and 65 years, normal or corrected hearing, no neurological disorder (e.g., epilepsy, tumor, lesion), and no earlier head or brain injury. We recorded RS EEG data of 38 participants from the general population. Based on standard data quality assessment, five participants were excluded due to remaining, excessive muscle and movement-related artifacts. Hence, 33 individuals (10 males, 22 females, 1 other; mean age = 23,36; SD = 2.65, range = 20–31) were selected for the current study. The sample consisted of university students (one recent graduate), who were either rewarded monetarily or by study credits. All participants provided written informed consent before participation.

### Data acquisition and pre-processing

Participants were invited to two sessions, a neuropsychological assessment, and the EEG recording. HP was assessed by the Launay-Slade Hallucination Scale (LSHS), a 16-item questionnaire designed to probe non-clinical hallucinatory predisposition (Larøi and Van Der Linden 2005; Launay and Slade 1981). For each participant, we obtained the total HP score as well as scores of the 5-item auditory HP (A-HP) and the 3-item auditory verbal HP (AV-HP) subscales following (Pinheiro et al. 2020). See Supplementary Material A for details.

Ten minutes (5 min eyes-open, EO; 5 min eyes-closed, EC; same order for all participants) of RS EEG data were recorded using a 128-channel actiCHAMP active system (Brain Products GmbH, Gilching, Germany). Electrodes were placed according to the international 10–20 system. The data were recorded at a sampling rate of 1000 Hz and FCz served as an online reference electrode while participants were sitting comfortably in an acoustically shielded EEG-booth. Electrode impedances were kept below 10 kΩ. Participants were asked to stay awake and to minimize body movements including blinking. EEG data were preprocessed using the MATLAB-based toolbox EEGLAB v2021 (Delorme and Makeig 2004) according to standard procedures for EEG RS data (Supplementary Material B). Only EC data were used for later data analyses as attention-induced alpha modulations are stronger in EC than in EO conditions (Wöstmann et al. 2020).

### Data preparation and feature extraction

We divided the dataset based on participants’ LSHS scores into two subsets: A “normative” set comprising the lower two-thirds of the total HP scores, (N = 26; 7 males, 18 females, 1 other), which we used to train the Brain State allocation model. This served to provide a normative and robust distribution of the dynamical features of the alpha fluctuations. The upper third of the total HP scores (N = 7; 3 males, 4 females) formed a “deviant” set, which was applied to the trained model to infer how the higher scores alter the distribution of the dynamical features. The splitting of the dataset was *not* to perform a group comparison but to obtain a realistic and robust model solution. Therefore, individual variability in state dynamics and HP of all participants was considered in the statistical analysis to account for the continuum perspective on HP. See Supplementary Material C for details on the splitting procedure.

**Fig. 1:**
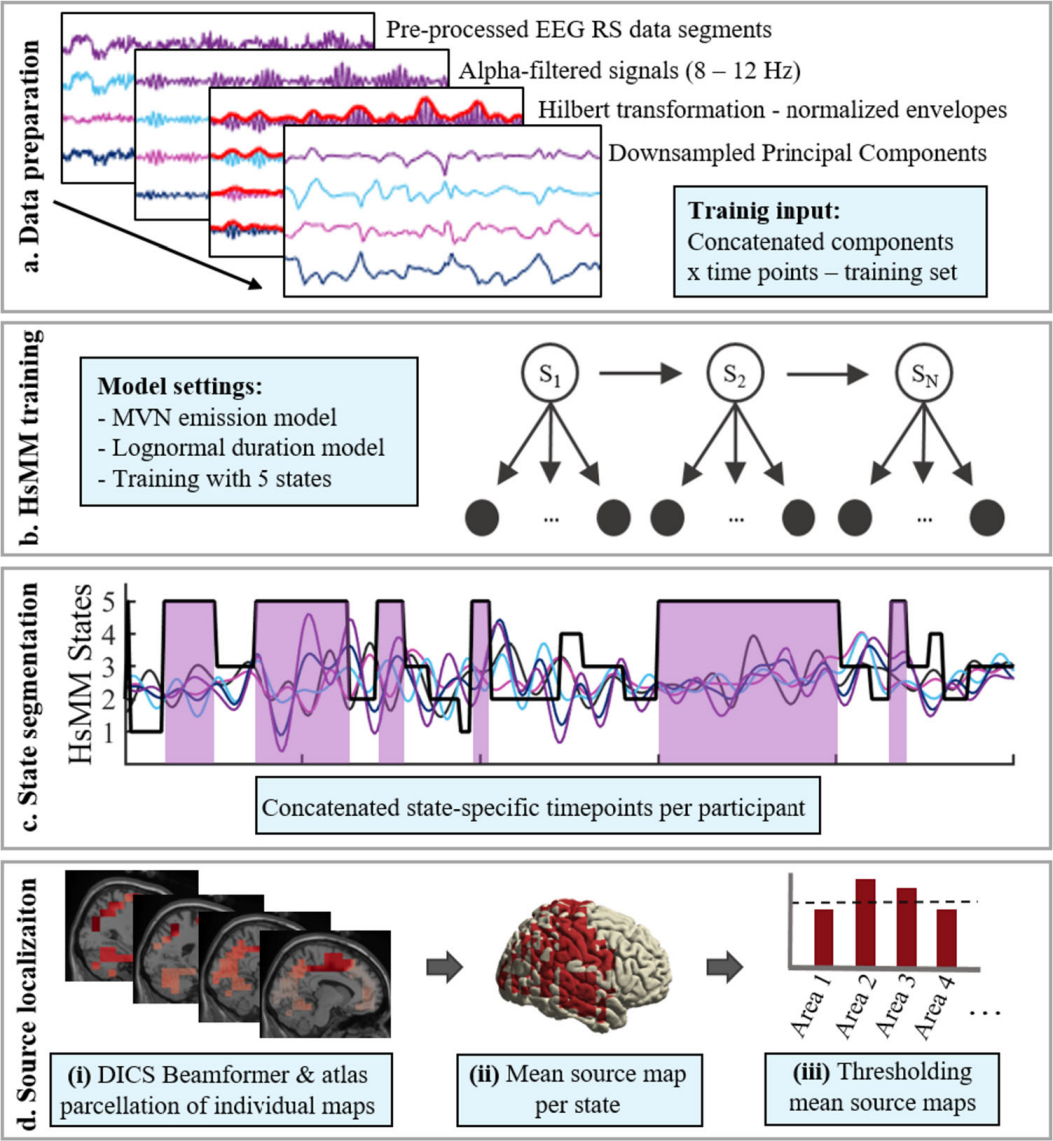
Schematic visualization of the analysis pipeline. **a** Data preparation. Pre-processed EEG RS data segments are filtered to the alpha band (8–12 Hz), Hilbert transformed, normalized, and log-transformed (log not shown here). Signals were then subjected to PCA with (30 components), downsampled to 64 Hz, and concatenated in time. **b** HsMM training. Concatenated data were trained using a Multivariate Normal (MVN) emission model and a lognormal duration model to decompose the data into 5 states **c** State segmentation. Data segments of each state were extracted and concatenated. **d** Source localization. (i) Data segments per state and participant were localized using the Dynamic Imaging of Coherent Sources (DICS) beamformer implementation in Fieldtrip. Sources were then parcellated into 116 areas using the Automated Anatomical Labeling (AAL) atlas (Tzourio-Mazoyer et al. 2002). (ii) The individual source activity maps were averaged across participants to create mean source maps of each state (iii) Mean source maps were z-transformed and thresholded to only keep the highest 25% of active sources

For data preparation prior to modeling, we followed standard HMM/HsMM data preparation pipelines (Baker et al. 2014), including envelope extraction of the alpha frequency (Supplementary Material D). See Fig. 1 for a schematic visualization of our pipeline, including major data processing and analysis steps.

### Dynamic Brain State allocation using a Hidden semi-Markov Model

We used the variational Bayes framework of the HsMM (Trujillo-Barreto et al. 2024). The HsMM is used to model fluctuations in the temporally concatenated RS EEG alpha envelope of all participants, in which a brain state is defined as recurrent and distinct periods of time during which the statistical properties of the data (mean and covariance) of the multichannel envelope are stable (Baker et al. 2014; Trujillo-Barreto et al. 2024). Accordingly, the state transitions mark the points at which the statistical properties of the data change and have been paralleled with the dynamic and systematic switching between underlying RSNs (Baker et al. 2014; Quinn et al. 2018). While the states are estimated at the group level, participant-specific post-hoc state metrics can be obtained directly from the state sequence. This provides insight into individual temporal dynamics of FC, which, in turn, allows between-participant comparisons.

The model was trained on the data of the 26 lower-scoring participants (i.e., the training set). The number of states was set to five for two main reasons: (i) Preliminary data screening of both individual and group-based HsMMs revealed that five states adequately characterize temporal state dynamics in the alpha band; (ii) Increasing the number of states may result in unreliable parameter estimates, which poses the risk of model overfitting (Trujillo-Barreto et al. 2024). Hence, given the ample between-participant variability and computational costs, a five-states HsMM was deemed a good compromise. We used a Multivariate Normal (MVN) distribution to model states’ emissions and a log-normal distribution to model the state durations. This implies that the hidden states generate normally distributed data at each time point with variable lengths following a log-normal distribution. The variational inference algorithm was randomly initialized and repeated ten times to avoid dependence on initial conditions (Trujillo-Barreto et al. 2024).

#### State sequence, calculation of state dynamics, and generation of state topographies

The state sequences of all individuals of the “normative” training set were directly obtained as the model output. The state sequences of the “deviant” test set were decoded by applying the already estimated model parameters from the training procedure to the unseen data, which results in a sequence of probabilities of each state being active at each time point. The state with the highest probability was chosen for the state sequence. The states’ fractional occupancy (FO: the total time occupied by a state compared to the total recording time) and mean duration (MD: average time a state is active) were extracted to describe the temporal dynamics per state and participant. State MD values were obtained by fitting a lognormal curve to the histogram of empirical state durations of each participant and state to extract the location (mu) and scale (sigma) parameters of the lognormal distribution. The lognormal mu values were then transformed to a normal scale to obtain interpretable duration values in milliseconds. The FO values were calculated by summing up each individual activation duration divided by the total recording time (i.e., 5 min) and are expressed as a percentage. The states' topographies were obtained by projecting the mean of the estimated emission distributions from the HsMM back to the sensor space. To this end, the mean vector of the estimated MVN distribution for each state was multiplied by the PCA coefficients obtained during the data preparation (Supplementary Material D).

### Source reconstruction of alpha Brain States

To assess whether the HsMM alpha states correspond to well-known RSNs, we used source localization of the original EEG data since the localization of envelope data is not directly interpretable. Spectral analyses and later source localization were performed using the MATLAB-based Fieldtrip toolbox (v20210914) (Baillet et al. 2011; Oostenveld et al. 2011). Source reconstruction of the participant-state data blocks in the frequency domain was carried out using the Dynamic Imaging of Coherent Sources (DICS) method (Gross et al. 2001) as implemented in Fieldtrip. DICS uses a spatial filter to detect and localize coherent sources, i.e., voxels that show functional synchrony, and is especially suited for the source reconstruction of oscillatory components in continuously recorded M/EEG signals (Drakesmith et al. 2013; Gross et al. 2001). The individual source images were parcellated using the Automated Anatomical Labeling (AAL) (Tzourio-Mazoyer et al. 2002) into 116 cortical and sub-cortical areas. The parcellated sources were then averaged across participants to obtain one mean source image per state. To assess the areas of highest source activation per state, the mean source images were z-transformed and thresholded to keep only source points exceeding the 75-th percentile (z-score = .675). The preparation of state-specific data segments and details of the source reconstruction are outlined in Supplementary Material E.

### Statistical analyses

Statistical analyses were performed in IBM Statistics SPSS v26 (IBM Corp., 2019). We tested for the predictive value of the Brain States’ dynamical features FO and MD for total HP and the two subscales, A-HP and AV-HP. Six hierarchical linear regression analyses were performed, two for each scale as a dependent variable (DV) with the states’ FO or MD as predictors. To find specific state dynamics that most accurately predict the DV, we used a backward elimination where the criterion for exclusion of predictors was set to the probability of F >/ = .1 in all models. We additionally computed bias-corrected accelerated (BCa) confidence intervals. The significance threshold for all statistical analyses was set at .05.

## Results

### Alpha Brain States' dynamical features

**Fig. 2:**
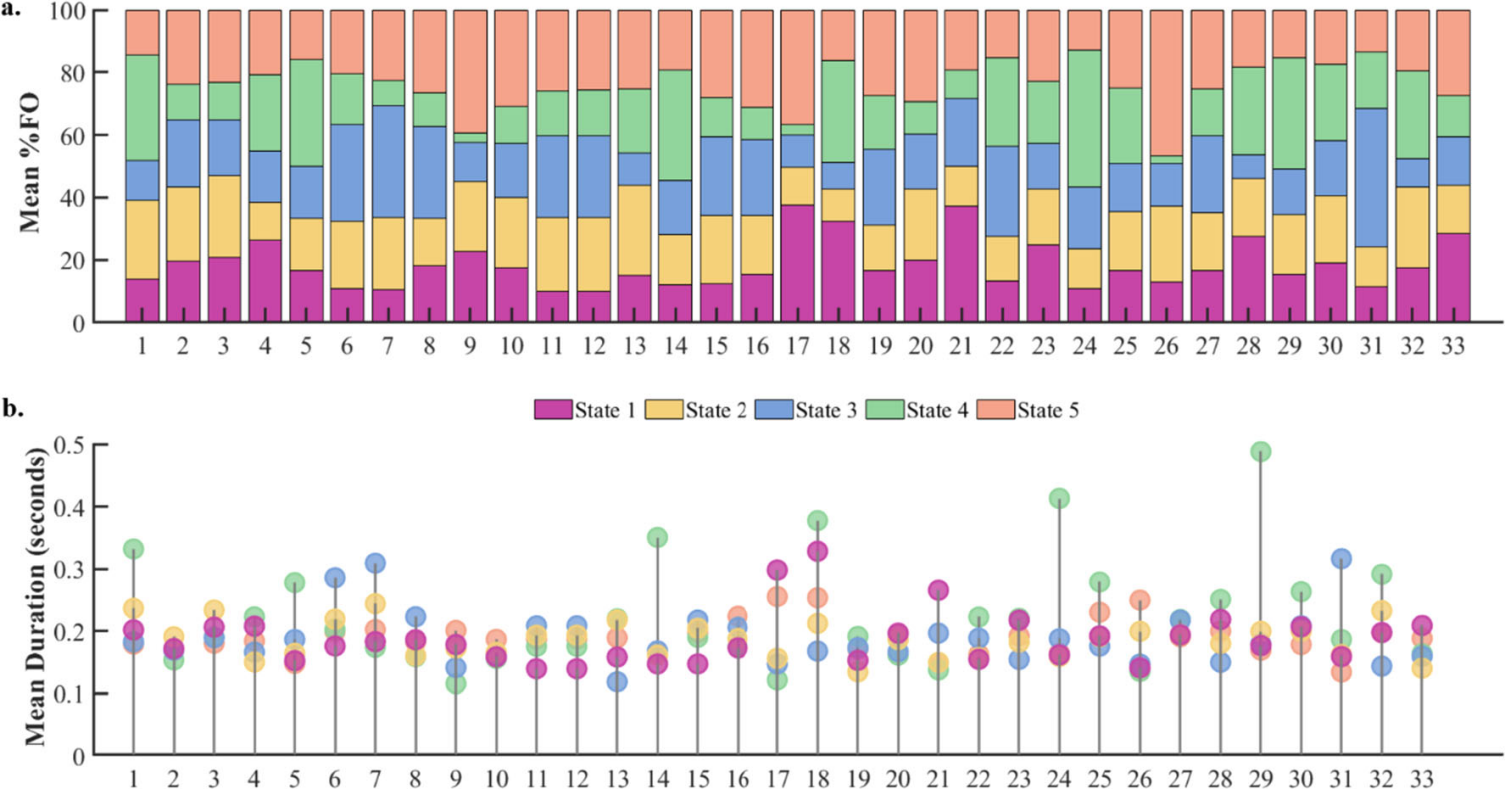
Fractional occupancy and mean duration of all states and participants. Distribution of Fractional Occupancy (FO, panel **a**) and mean duration (panel **b**) of all participants. Participants are ordered according to their total hallucination proneness (HP) scores from low to high. FO is provided in percentage (%). Durations are given in seconds

**Fig. 3:**
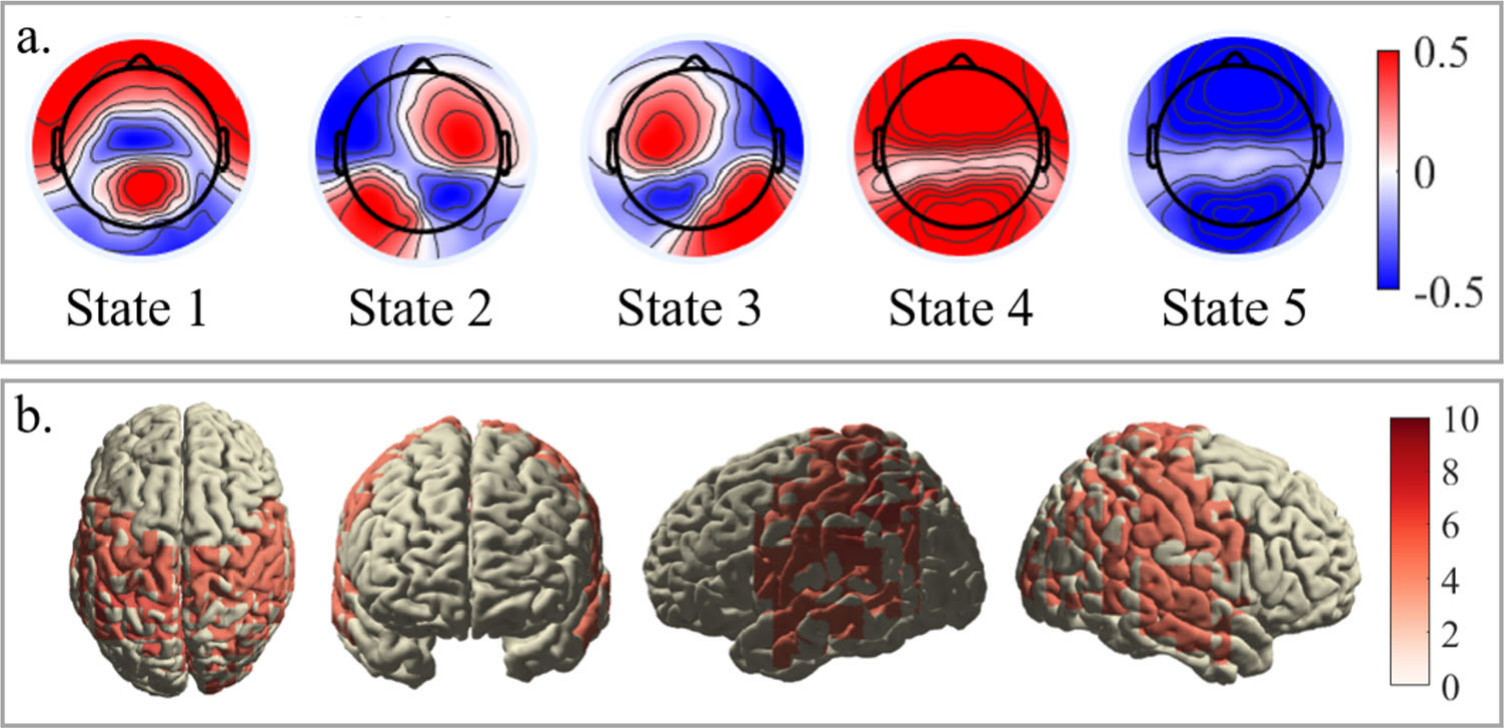
State topographies and source activation of state 1. **a** The maps depict the most representative topography for each state, obtained by multiplying the mean vector of the estimated Multivariate Normal (MVN) distribution of each state with the Principal Component Analysis (PCA) coefficients obtained during the data preparation The order of the states is arbitrary. The scale of the state maps depicts microvolts. **b**. Cortical distribution of state 1 active sources. Activation is expressed as Neural Activity Index (NAI) (Van Veen et al. 1997). Images were generated by (i) parcellating the individual source images using the Automated Anatomical Labeling (AAL) atlas (Tzourio-Mazoyer et al. 2002) into 116 areas, (ii) averaging the activity within each parcel across participants, (iii) thresholding the mean sources images based on the 75-th percentile of source activity (> / = z-value of .675), and (iv) interpolating the active sources onto a template brain mesh

Figure 2 shows FO and MD for each participant in panels A and B, respectively. All five states were represented in all participants, but their FO and MD showed considerable between-participant variability. For instance, state 4 showed mean durations of up to .5 s, while the durations of the other states are mostly clustered around .2 s. Figure 3 panel A depicts the state maps, i.e., the most representative topography for each state. The order of states is arbitrary. State 4 and 5 showed a similar activity distribution, although with opposite polarity, which may indicate complementary states of high and low alpha power.

### Results of the statistical analyses

Assumptions such as normality of residuals, linearity, and homoscedasticity for the regression models for HP and A-HP as DV were met. For the models with AV-HP as DV, the residuals were not normally distributed, which may compromise the generalizability of the results. The results of the final models (i.e., after the exclusion of insignificant predictors) are summarized in Table 1. The complete results of all regression models including all steps of the backward elimination procedure can be found in Supplementary Material G. The stepwise regression with A-HP as DV resulted in a model with only state 1 FO as significant predictor F(1,32) = 7.818, *p* = .009, which accounted for 20.1% of the variation in A-HP (R^2^ = .201). Similarly, the model with A-HP as DV and duration values as predictors indicated state 1 MD as only significant predictor F(1,32) = 5.949, *p* = .021, accounting for 16.1% of the variation (R^2^ = .161). Further, the final regression model with AV-HP as DV also revealed state 1 FO as significant predictor, F(1,32) = 5.219 *p* = .029, which accounted for 14.4% of the variation in AV-HP (R^2^ = .144). Finally, the MD of state 1 significantly predicted AH-HP, F(1,32) = 5.070 *p* = .032, explaining 14.1% of the variation in AH-HP (R^2^ = .141). The two regression models with HP as DV did not reveal any significant predictors.

**Table 1:** Summary of significant regression model results

Pred	Coefficients					Model summary			
	DV	B	B CI	β	t	F	R^2^	Adj. R^2^	*p*
S1 FO	A-HP	.210	[.065 .417]	.449	2.796**	7.818	.201	.176	.009**
S1 MD	A-HP	37.607	[6.160 69.053]	.401	2.439*	5.949	.161	.134	.021*
S1 FO	AV-HP	.134	[.013 .255]	.375	2.252*	5.070	.141	.113	.032*
S1 MD	AV-HP	23.623	[.029 44.712]	.380	2.285*	5.219	.144	.116	.029*

Each row contains the regression coefficients and model summary for each final model, i.e., after stepwise exclusion of insignificant predictors

Pred., predictor variable; S1, state 1; DV, dependent variable; FO, fractional occupancy; MD, mean duration; A-HP, auditory hallucination proneness; AV-HP, auditory-verbal hallucination proneness

N = 33; *p < .05; **p < .01

In summary, state 1 FO and MD significantly predicted both A-HP and AV-HP, but not general HP. This means that individuals scoring higher on the auditory and auditory-verbal subscales of the LSHS showed greater occupancy and longer duration of state 1. This might show an effect specific to the auditory modality of hallucinatory experiences. The explained variance of the final models ranged between 14.1 and 20.1%. These results were mostly confirmed by the more robust bootstrapping procedure and corresponding BCa confidence intervals, which are summarized in Table 2.

**Table 2:** Bootstrapping results for significant regression coefficients

					BCa 95% CI	
Pred	DV	B	Std. error	*p*	Lower	Upper
S1 FO	A-HP	.241	.089	.009**	.062	.417
S1 MD	A-HP	37.607	16.597	.018*	14.402	84.536
S1 FO	AV-HP	.134	.063	.039*	.000	.280
S1 MD	AV-HP	23.623	13.604	.062	5.184	62.618

Each row contains the bootstrapping results of significant regression coefficients of the final models i.e., after stepwise exclusion of insignificant predictors, including the bias-corrected accelerated (BCa) 95% confidence interval (CI)

Pred., predictor variable; S1, state 1; DV, dependent variable; FO, fractional occupancy; MD, mean duration; A-HP, auditory hallucination proneness; AV-HP, auditory-verbal hallucination proneness. Results are based on 2000 bootstrap samples

N = 33; *p < .05; **p < .01

### Results of the source localization

All states' highest active sources were localized in the posterior part of the brain, spanning the superior and inferior parietal cortex, cingulum, the cuneus and precuneus, parts of the operculum and of the occipital cortex. Amongst others, state 1 was characterized by source activity in the posterior cingulate cortex (PCC), precuneus, and cuneus, which constitute major hubs of the DMN. Additionally, state 1 sources were found in left and right postcentral gyrus as well as superior and middle temporal gyrus in both hemispheres, corresponding to the somatosensory and auditory networks, respectively (Fig. 3 panel B). See Supplementary Material H for the mean source images of states 2–5 and the labels of all active areas surviving the thresholding per state.

## Discussion

Hallucinatory experiences are spontaneous, transitory, and dynamic false percepts that arise as a result of self-monitoring and source attribution difficulties (Blakemore et al. 2000; Brookwell et al. 2013). HP is associated with heightened sensory sensitivity, which, in turn, is linked with fluctuations in the alpha frequency band (Blakemore et al. 2000; Craddock et al. 2017; Lewis-Hanna et al. 2011; Shen et al. 2019). This study investigated if RS alpha brain state dynamics predict non-clinical HP, A-HP, and AV-HP. To this end, five brain states and their temporal dynamics (FO and MD) were estimated with a HsMM. Using frequency-domain source reconstruction, we further localized the sources of each state. The results showed that MD and FO of a state corresponding to somatosensory, auditory, and posterior DMN areas (here state 1) predicted individual differences in A-HP and AV-HP, but not general HP. State 1 may reflect an attentional bias toward internally generated events and heightened awareness of and sensitivity for auditory sensations. Together, this could suggest increased vulnerability to hallucinatory experiences in the auditory domain.

### Resting state alpha dynamics might modulate sensory sensitivity to internal stimuli

Consistent with the high between-participant variability in state dynamics observed in the current study, previous research found that the resting brain engages in stimulus-independent activities like mind-wandering and autobiographical memory retrieval with inter-individual differences in shifting patterns and preferences for a given cognitive mode, or “phenotype” (Diaz et al. 2013; Raichle 2015). These so-called RS phenotypes are characterized by distinct electrophysiological signatures of cognition and behavior (Pipinis et al. 2017; Tarailis et al. 2021). The time-varying FC signatures characterized by the HsMM state sequence could thus reflect shifts between different cognitive modes during rest that might coincide with modulations in attention between specific sensory modalities.

In cognitive tasks, alpha activity is linked to selective attention, cognitive control, and perceptual sensitivity (Hindriks et al. 2017; Klimesch et al. 2007). Other studies found that pre-stimulus alpha power is inversely related to perceptual awareness but does not predict discrimination accuracy (Benwell et al. 2022; Benwell et al. 2017). Similarly, it was suggested that alpha power fluctuations link to changes in neural baseline excitability and thus influence neural responsiveness to both signal and noise (Iemi and Busch 2018). In turn, alpha dynamics may reflect criterion changes in an individual’s response behavior (i.e., heightened neural excitability leading to more liberal responses), rather than perceptual accuracy (Iemi and Busch 2018). Alpha activity modulations, especially in EC conditions, were further suggested to reflect changes in auditory attention through the inhibition of irrelevant, non-salient sensory information (Strauß et al. 2014; Wöstmann et al. 2020). These findings support the hypothesis that alpha dynamics shape the allocation of attentional resources and the processing of sensory inconsistencies (Jensen et al. 2012). However, most of these findings were task-related and are thus not necessarily transferable to spontaneous, RS alpha fluctuations. Although a direct effect of alpha modulations on cognition is difficult to show in pure RS conditions, evidence supports the role of alpha in attention, cognitive control, and perceptual stability (Braboszcz and Delorme 2011; Katyal et al. 2019; Mahjoory et al. 2019; Mathewson et al. 2009; Sadaghiani and Kleinschmidt 2016). Given that the identified HsMM states reflect recurrent signatures of alpha activity independent of task demands, the states' temporal dynamics could inform about spontaneous shifts in attention from external to internal stimuli and perceptual sensitivity thereof, rather than perceptual accuracy per se (Craddock et al. 2017; Klimesch et al. 2007; Wöstmann et al. 2020).

The current results suggest that specific RS alpha dynamics (FO and MD of state 1) reveal individual differences in auditory HP. This relationship may be driven by alpha modulations of neural excitability (Iemi and Busch 2018). Heightened baseline excitability is thought to amplify both signal and noise, which may alter an individual’s tendency to “detect” stimuli in the absence of sensory stimulation and confuse internally generated sensory events as coming from an external source (Iemi and Busch 2018; Ilankovic et al. 2011; Northoff and Qin 2011; Stephane et al. 2010). Hallucinating and hallucination-prone individuals indeed show increased difficulties in source and reality monitoring and an external attribution bias irrespective of a clinical diagnosis (Blakemore et al. 2000; Brookwell et al. 2013; Levine et al. 2004). Interestingly, the relationship with state 1 FO and MD was only found for the auditory and auditory-verbal modality of hallucinatory experiences but not for general HP. This raises the question of whether alpha dynamics not only reflect fluctuations in attention and neural responsiveness to internally generated information, but also how attentional resources are distributed between sensory modalities (Keller et al. 2017).

### Alpha HsMM state sources and their functional significance for hallucination proneness

The states’ source localization results further corroborate earlier findings that linked alpha activity to posterior hubs of the DMN (Hindriks et al. 2017; Mantini et al. 2007). Likewise, the current alpha HsMM states show similarities with a “posterior higher-order cognitive” state identified by Vidaurre et al. (2018), associated with posterior nodes of the DMN and high coherence and power in the alpha frequency. Of note is that their approach considerably differs from the current one, in that they used time-domain source-reconstructed MEG data as input to a time-delay embedded Hidden Markov Model (TDE-HMM). The TDE-HMM allows identification of both spectrally and temporally resolved state characteristics. However, both approaches confirm that spontaneous brain activity is organized into short-lived recurrent brain states with specific spectral information and spatial correspondence to well-known RSNs.

The source localization of state 1 specifically revealed simultaneously active sources in bilateral pre-, para-, and post-central lobes, bilateral inferior and superior parietal lobes, bilateral (pre-)cuneus, bilateral PCC, and bilateral superior and middle temporal lobes. These regions are associated with the somatosensory network, the auditory network, and posterior hubs of the DMN (Damoiseaux et al. 2006; Raichle 2015), suggesting that HsMM brain states associate with a mixture of RSNs (Chen et al. 2016; Hunyadi et al. 2019).

The active sources underlying state 1 could inform about brain areas and networks that express a heightened vulnerability to hallucinatory experiences. Michael et al. (2022) found that spontaneous oscillatory activity in somatosensory cortices reflects individual differences in bodily awareness. Further, subjective somatosensory experiences in the absence of external stimulation are thought to arise due to heightened levels of focused attention (Bauer et al. 2014), which in turn, is likely mediated by alpha power fluctuations (Benedek et al. 2014). One may speculate that such activity not only manifests in distorted bodily perception but potentially also in hallucinatory experiences in other sensory domains (e.g., auditory), particularly if paired with spontaneous activation of the auditory network. Activation of the superior and middle temporal lobes is commonly associated with the processing of incoming auditory stimuli. However, these activation patterns could be altered in individuals with auditory hallucinations due to a reduced signal-to-noise ratio of incoming sensory stimulation and abnormally elevated resting activation of the auditory cortex (Northoff and Qin 2011). In fact, abnormal untriggered engagement of the primary and secondary auditory cortices has been related to the experience of AVH (Kompus et al. 2011; Northoff and Qin 2011). The DMN has been associated with spontaneous, task-independent cognition, including mind-wandering and daydreaming (Raichle 2015). The PCC, a posterior hub of the DMN, is thought to play a key role in internally directed cognition, autobiographical memory, and reorienting attention. Further, functional interactions between the PCC and other networks are crucial for conscious awareness and perception (Leech and Sharp 2014). Along with aberrant auditory cortex activation, many studies reported abnormally elevated DMN activation in individuals with AVH, which may indicate an attentional bias toward internally generated (auditory) events and a failure in downregulating auditory processing systems (Kompus et al. 2011; Northoff and Qin 2011). Schizophrenia patients with AVH further show increased connectivity between posterior parts of the DMN and regions associated with auditory processing, which may contribute to the misattribution of self-generated sensory events to an external source, and thus the experience of auditory hallucinations (Mannell et al. 2010; Northoff and Qin 2011). Similarly, an fMRI network analysis revealed that aberrant activation of the DMN and the auditory network links to hallucinatory vulnerability in non-psychotic individuals (van Lutterveld et al. 2014), suggesting that changes in DMN dynamics are not unique to the clinical population but may serve as an early marker of increased HP in the general population. Lastly, Kottaram et al. (2019) applied an HMM to fMRI BOLD hemodynamics to investigate differences in RSN dynamics between patients with schizophrenia and healthy control participants. They found that patients spent a significantly shorter proportion of time in a state characterized by high DMN and low sensory network activation, however, once visited, the duration of that state was significantly longer as compared to the controls. These dynamics further correlated with the severity of positive symptoms, including hallucinatory experiences.

In summary, the current results suggest that non-clinical A-HP and AV-HP link to changes in alpha temporal dynamics of a state that is characterized by somatosensory, auditory, and DMN activation. Thus, individuals who are prone to auditory (verbal) hallucinatory experiences spend longer time segments in a state that may reflect an increased attentional bias toward internal events, potentially as they are more salient by default (Kapur 2003), combined with heightened neural responsiveness to (internally generated) auditory percepts. These results are consistent with findings that linked hallucinatory experiences with altered DMN-auditory network dynamics that, in turn, contribute to the well-established source and reality monitoring difficulties along the HP continuum (Allen et al. 2006; Brookwell et al. 2013; Northoff and Qin 2011; van Lutterveld et al. 2014).

### Limitations and future directions

Given that this study’s objective was to investigate the predictive value of alpha RS dynamics for non-clinical HP, the generalizability of the current findings to the clinical population is limited. Nevertheless, the continuity of psychosis-like symptoms and corresponding increased clinical risk has gained considerable weight through different methodological approaches (Allen et al. 2006; Badcock and Hugdahl 2012; Kusztrits et al. 2021). The current study thus contributes to a better understanding of how electrophysiological correlates can display possible changes in participants along the postulated HP continuum and might therefore critically inform early risk assessment of vulnerable individuals. However, is important to note that a single continuum may not fully explain the heterogeneity of neurocognitive changes and phenomenological characteristics in non-clinical and clinical AVH (Badcock and Hugdahl 2012; Corona-Hernández et al. 2022). Thus, the question arises whether some qualitative differences of the hallucinatory experiences between clinical and non-clinical populations can be explained by the same continuum of neural changes or whether so-called quasi-dimensional models of psychosis better account for this heterogeneity (Baumeister et al. 2017). Future research should therefore explore whether similar changes in RS temporal dynamics are also characteristic of individuals with AVH in the non-clinical and clinical spectrum.

The modeling of temporal dynamics in the current study was restricted to the alpha band, following its role in selective attention, cognitive control, and perceptual sensitivity, as well as its association with the DMN (Craddock et al. 2017; Hillebrand et al. 2012; Hindriks et al. 2017). This narrow-band filtering approach may have influenced the spatial overlap between states in the source space. Broadband data may reflect a richer repertoire of the electrophysiological signatures of the underlying RSNs operating within different frequency bands (Mantini et al. 2007). Thus, the current approach yields a specific view on RS dynamics through the lens of the alpha band frequency. Future studies could therefore explore how brain state temporal dynamics enfold in broadband data and how they differ between frequency bands. This could offer further insights into frequency-specific RS correlates of cognition and behavior.

Lastly, it is important to note that the current focus was on temporal dynamics and key sources of alpha brain states as a function of HP. Accordingly, the chosen approach revealed simultaneously and highly active sources of each state but did not provide evidence for functionally connected large-scale brain networks. Future research should therefore aim to unveil interaction between (sub-)network dynamics along the whole HP continuum, e.g., by using adaptations of the H(s)MM method with a multivariate autoregressive (MAR) emission model that model effective connectivity based on both amplitude and phase dynamics (Hernandez et al. 2022; Vidaurre et al. 2016).

In conclusion, the HsMM can be used to characterize narrow-band alpha RS fluctuations on a sub-second timescale and derive meaningful neurophysiological correlates of the HP continuum. Our findings suggest that an increased attentional bias towards and increased sensory sensitivity to internally generated auditory events, as it is often found in clinical voice-hearers, might already be characteristic of non-hallucinating but high-hallucination-prone individuals.

## Supplementary Information

Below is the link to the electronic supplementary material.

Supplementary file1 (DOCX 2726 KB)

## Data Availability

The software used for HsMM implementation in this manuscript is available at https://github.com/daraya78/BSD. Anonymized data will be shared with other researchers upon reasonable request to the corresponding author.
