# Supplementary material for: EEG resting state alpha dynamics predict an individual’s vulnerability to auditory hallucinations
Source: Cogn Neurodyn. 2024 Mar 22;18(5):2405–17. doi: 10.1007/s11571-024-10093-1 (PMC11564481; doi:10.1007/s11571-024-10093-1)
Supplement: Supplementary file 1 — Supplementary file1 (DOCX 2726 KB) [file 11571_2024_10093_MOESM1_ESM.docx]

**Supplementary Material and Methods**

**Title:** EEG resting state alpha dynamics predict an individual’s vulnerability to auditory hallucinations

**Journal name:** Cognitive Neurodynamics

**Authors:** Honcamp, H.^a*^, Duggirala, S.X.^a,e^, Rodino Climent, J. ^b^, Astudillo, A.^c^, Trujillo-Barreto, N.J.^d^, Schwartze, M.^a^, Linden, D.E.J.^e^ , van Amelsvoort, T.A.M.J. ^e^, El-Deredy, W.^f^, Kotz, S.A.^a*^

**Institutional affiliations:**

^a^Department of Neuropsychology and Psychopharmacology; Faculty of Psychology and Neuroscience, Maastricht University, the Netherlands

^b^Brain Dynamics Laboratory, Universidad de Valparaíso, Chile

^c^NICM Health Research Institute, Western Sydney University, Penrith, New South Wales, Australia

^d^School of Health Sciences, University of Manchester, Manchester, United Kingdom

^e^Department of Psychiatry and Neuropsychology, School of Mental Health and Neuroscience, Maastricht University Medical Center, Maastricht, the Netherlands

^f^Centro de Investigación y Desarrollo en Ingeniería en Salud, Universidad de Valparaíso, Chile

***Corresponding author:**

Hanna Honcamp
ORCID-ID: 0009-0001-3373-9368
Dept. Neuropsychology and Psychopharmacology
Maastricht University
Universiteitssingel 40, 6229 ER Maastricht, the Netherlands
Email: [h.honcamp@maastrichtuniversity.nl](mailto:h.honcamp@maastrichtuniversity.nl)

1. **Data acquisition – Behavioral data**

All items on the Launay-Slade Hallucination Scale (LSHS) (Larøi & Van Der Linden, 2005; Launay & Slade, 1981) were answered on a 5-point Likert scale (0 = “Certainly does not apply to me”, 1 = May not apply to me”, 2 = “Unsure”, 3 = May apply to me”, 4 = “Certainly applies to me”). The total score (sum of all scores on each individual item) ranges between 0-64, with higher scores indicating higher hallucination proneness (HP). Supplementary Table 1 and Fig. 1 show the descriptive statistics and the distribution of total HP, auditory HP (A-HP), and auditory verbal HP (AV-HP) for all participants of the current sample, respectively.

**Suppl. Table 1 Descriptive statistics of hallucination proneness scores**

|  | **Mean** | **SD** | **Range** |
| --- | --- | --- | --- |
| HP | 15.36 | 10.68 | 0 - 42 |
| A-HP | 4.33 | 4.04 | 0 - 14 |
| AV-HP | 2.09 | 2.68 | 0 - 8 |

Hallucination proneness (HP); auditory HP (A-HP); auditory verbal HP (AV-HP); N = 33.


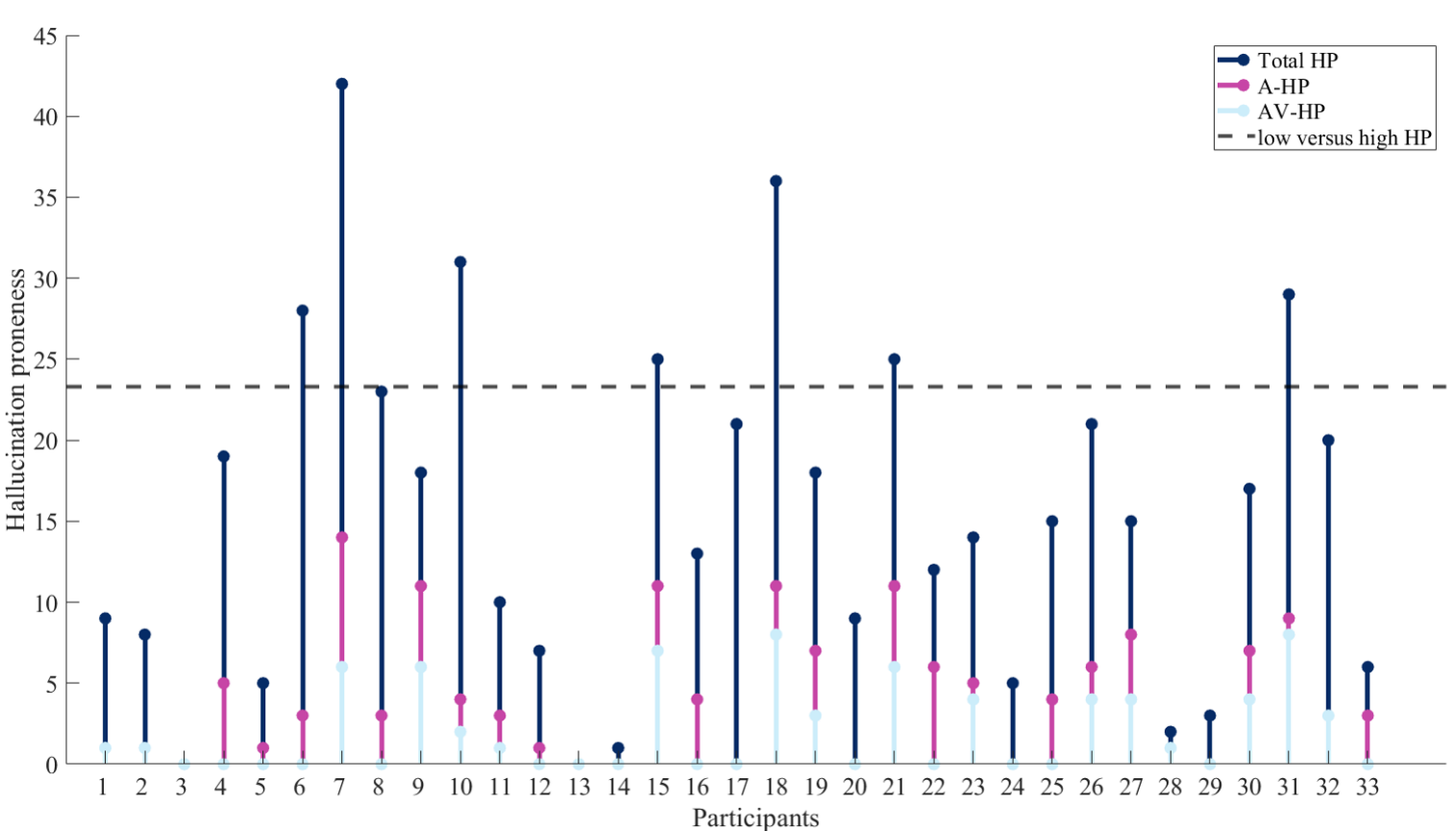


**Suppl. Fig. 1 Distribution of hallucination proneness, auditory hallucination proneness, and auditory verbal hallucination proneness**

The figure shows the distribution of hallucination proneness (HP), auditory HP (A-HP), and auditory verbal HP (AV-HP) scores in the current sample. The individual participants (1-33) are plotted on the x-axis against their scores on the Launay-Slade Hallucination Scale (LSHS). The dashed line indicates the splitting between ”lower” and ”higher” scoring participants based on their total HP scores. Thus, participants above the dashed line correspond to the upper third of the total HP scores.

1. **EEG RS data pre-processing**

The continuous resting state (RS) EEG data were downsampled to 512 Hz and filtered between 1-40 Hz with a finite impulse response (FIR) band-pass filter. Subsequently, the default EEGLAB procedure (clean_rawdata, flat-line criterion = 5; channel criterion = 0.8; line noise criterion = 4) for bad channel detection was used to show and reject noisy channels. On average, 6.39 (SD = 5.208) channels were rejected for each participant. Rejected channels were replaced using spline interpolated neighboring channels. Finally, data were re-referenced to the average of all channels. Automatic Subspace Reconstruction (ASR) with a burst criterion of 20 was applied to remove remaining non-stationary artifacts. Lastly, combined PCA/ICA was applied to the eyes-open and eyes-closed data separately to control data rank deficiency and reduce dimensionality. Thus, each individual data set was decomposed into 20 independent components. Components being i) eyeblinks, ii) horizontal and vertical eye movements, iii) remaining noisy channel activity, iv) muscle-related activity, and v) line noise were rejected. On average, 4.64 (SD = 1.03) components were rejected per dataset. Only the eyes-closed data was used for further analysis.

1. **Training and test datasets**

This splitting between a “normative” (N = 26) and “deviant” (N = 7) set was based on two considerations. First, we aimed to show a set of states that reflects normative FC signatures of the alpha RS to estimate the states’ temporal dynamics. We expected neither anatomical nor corresponding scalp-topographical differences in FC signatures between lower- and higher-prone individuals so that the same topographical state maps could be used for all participants. Second, the HsMM is tuned to find the best-fitting states based on the statistical properties of the input time series. If individuals on the upper end of the HP continuum would show alterations in state switching behavior (consistent with our expectations), performing the model training on all (N = 33) participants could introduce a bias in the estimation of the states’ dynamics parameters, thereby conflating “normative” and “deviant” dynamics. We refer to the normative and deviant set as training and test set, respectively.

Splitting data into a training set and a test/decoding set is a common modeling approach to model the extracted features of several data sets (Murphy, 2012). Such methods have been applied to RS EEG data in various clinical contexts (Chen, Lu, Xie, & Shang, 2020; de Miras, Ibáñez-Molina, Soriano, & Iglesias-Parro, 2023; Huggins et al., 2021; Mao, Zhu, Li, Zhang, & Sun, 2018; Xu, Zheng, Mao, Wang, & Zheng, 2020). Moreover, a similar splitting of the LSHS scores into higher and lower scoring individuals was used in prior studies (Collignon, Van der Linden, & Larøi, 2005; Garrison, Moseley, Alderson-Day, Smailes, Fernyhough, & Simons, 2017; Kanemoto, Asai, Sugimori, & Tanno, 2013; Larøi, Van der Linden, & Marczewski, 2004)).

1. **Data preparation for modeling**

*Training set*

The data processing was performed in MATLAB v2020b, using the Brain Dynamics Toolbox and custom scripts (Trujillo-Barreto, Araya, Astudillo, & El-Deredy, 2024). To prepare the data of the training set, the following steps were applied for each participant separately. The data were first band-pass filtered to the canonical alpha frequency (8-12 Hz) and linearly detrended. Subsequently, the envelope (i.e., the size of the analytic signal) was extracted using Hilbert transformation and then normalized by the global standard deviation across channels per participant. The latter ensured that any HsMM state would not be driven by differences in amplitude between participants but rather by the temporal dynamics of the data. The data were then logarithmically transformed. To increase computational efficiency, while preserving most of the information, the training data were temporally concatenated, its dimensionality reduced using 30 principal components, keeping 90.125% of the explained variance, and downsampled to 64 Hz.

*Test set*

Data of the test set were then transformed accordingly, except that the coefficients obtained from the PCA of the training set were used to decompose the test data. This prevented data leakage while ensuring that data of both sets were transformed into the same dimensional space to allow valid comparisons of state dynamics across all participants.

1. **Technical details of the Dynamic Imaging of Coherent Sources** (**DICS) beamformer approach for source reconstruction**

*Preparation of state-specific data segments*

First, the cleaned RS EEG data were filtered to the alpha band (8-12 Hz) to match the training frequency band. Then, the state sequence (training and test sets; N = 33), concatenated across all participants, was upsampled to match the data sampling frequency after preprocessing/cleaning and prior to data preparation for modeling (from 64 Hz to 512 Hz). We chose to upsample the state sequence instead of downsampling the data to keep as many data points as possible to ensure robust source reconstruction. The pre-processed data were then segmented according to the upsampled state sequence into state-specific data points for each participant. For each state, the data points of all participants were then standardized and concatenated, resulting in 165 participant-state (33*5) data blocks.

*Source reconstruction*

We employed a template head (volume conductor) model, normalized to Montreal Neurological Institute (MNI) space, provided by Fieldtrip. The head model was generated using the boundary element method (BEM) (Fuchs, Kastner, Wagner, Hawes, & Ebersole, 2002) and held meshes for the scalp, skull, and brain with conductivity values of 0.33, 0.0041, and 0.33, respectively. The electrode layout with 128 channels was manually co-registered to the head model using an interactive function in Fieldtrip. The source model was manually generated and contained 2015 vertices spanning the source space with 10 mm resolution. The lead field (i.e., the forward solution), yielding an estimation of how the currents spread from dipolar sources to the sensors, was calculated accounting for the head model and the different tissue conductivities, the pre-defined source space, and aligned electrode configuration.

Multitaper frequency transformation was applied to the full length of each data segment to compute the cross-spectral density (CSD) of the 10 Hz frequency component using a discrete prolate spheroidal sequences (DPSS) taper with a frequency smoothing of 2 Hz. The Dynamic Imaging of Coherent Sources (DICS) uses a spatial filter to detect and localize coherent sources, i.e., voxels that show functional synchrony (Drakesmith, El-Deredy, & Welbourne, 2013). In contrast to time-domain beamformer methods, DICS is based on frequency domain data, i.e., the estimation of source coupling relies on the CSD of all sensor pairs. The beamformer output yields a spatial distribution of source power in a chosen frequency band (here alpha). CSD computation and beamformer projection were performed separately for each participant-state data block with regularization of 5%, which is a commonly used regularization strength in the literature and recommended in Fieldtrip. Finally, the beamformer output was normalized by an estimate of the source-projected noise resulting in the Neural Activity Index (NAI) (Van Veen, Van Drongelen, Yuchtman, & Suzuki, 1997).

1. **Additional results of alpha brain** **states dynamical features**


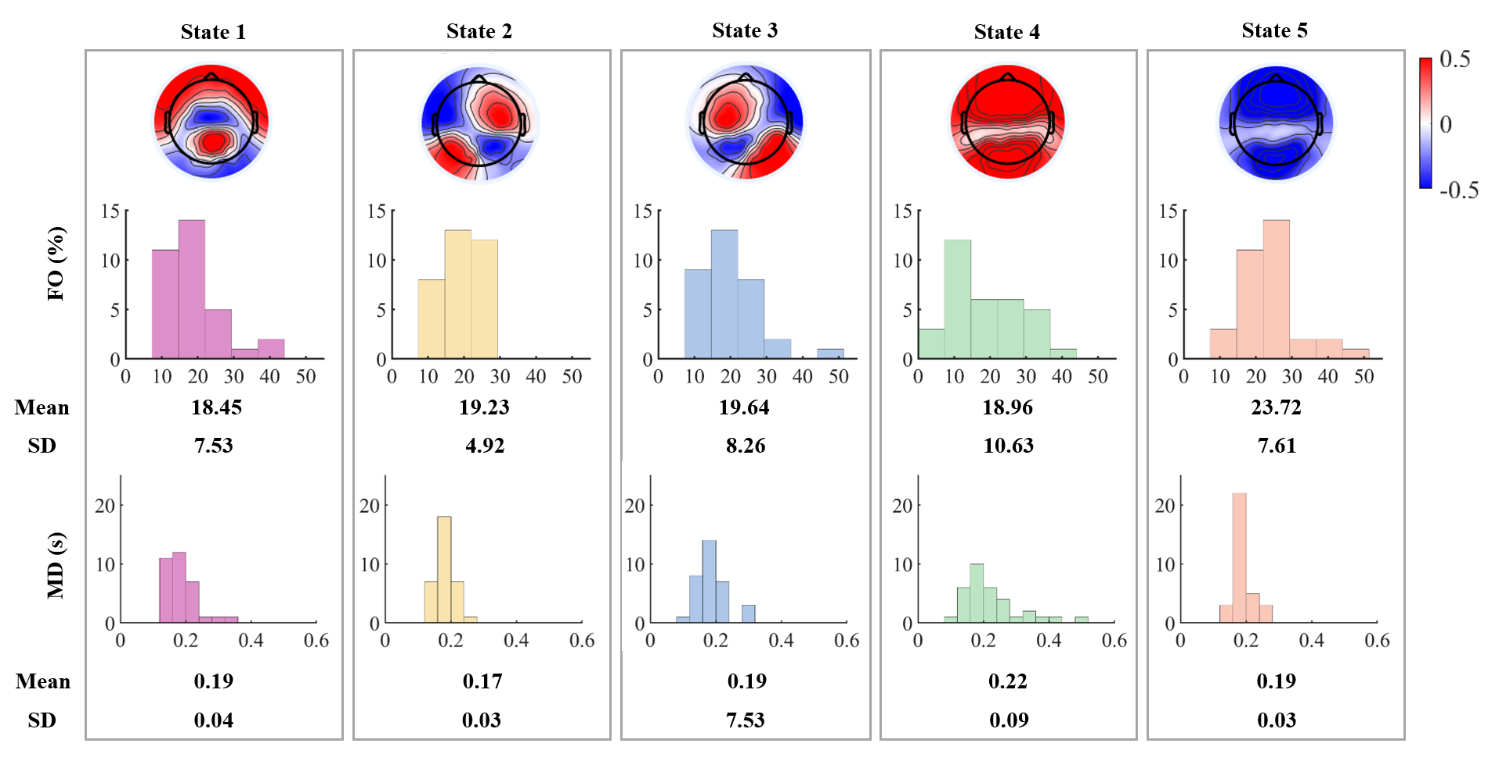
**Suppl. Fig 2 Histograms of fractional occupancy and mean duration of all states across participants**

The upper and lower panels show histograms of fractional occupancy (FO) in percent (%) and mean duration (MD) in seconds (s) per state, respectively. The scale of the state maps indicates microvolts. The mean and standard deviation (SD) values refer to the mean and SD of FO or MD values per state across all participants.

1. **Complete results of the hierarchical linear regression models**

This section contains the complete results of the regression analyses. To assess the predictive value of any of the state’s fractional occupancy (FO) and mean duration (MD) values for general HP and the two subscales, A-HP and AV-HP, we performed hierarchical linear regression analyses. We created six regression models: i) HP as dependent variable (DV) and FO values of state 1-5 as predictors, ii) HP as DV and MD values of state 1-5 as predictors, iii) A-HP as DV and FO values of state 1-5 as predictors, iv) A-HP as DV and MD values of state 1-5 as predictors, v) AV-HP as DV and FO values of state 1-5 as predictors, and finally vi) AV-HP as DV and MD values of state 1-5 as predictors. For all models, we used a backward exclusion procedure in which non-significant predictors were removed stepwise according to the following criterion: Probability of F-to-remove >/= 0.1. The significance threshold for all statistical analyses was set at .05.

**Suppl. Table 2 General hallucination proneness as dependent variable and fractional occupancy values of states 1-5 as predictors.**

|  | **Coefficients** | | | **Model summary** | | | |
| --- | --- | --- | --- | --- | --- | --- | --- |
| **Predictor** | **β** | **t** | **p** | **F** | **R^2^** | **Adj. R^2^** | **R^2^**Δ |
| **Step 1** |  |  |  | .581 | .077 | -.055 | .077 |
| (Constant) | - | 1.821 | .079 |  |  |  |  |
| State 1 FO | -.077 | -.283 | .779 |  |  |  |  |
| State 2 FO | -.281 | -1.151 | .259 |  |  |  |  |
| State 3 FO | -.189 | -.796 | .433 |  |  |  |  |
| State 5 FO | -.25 | -.128 | .899 |  |  |  |  |
| **Step 2** |  |  |  | .797 | .076 | .11 | -.001 |
| (Constant) | - | 1.849 | .075 |  |  |  |  |
| State 1 FO | -.086 | -.330 | .744 |  |  |  |  |
| State 2 FO | -.292 | -1.30 | .204 |  |  |  |  |
| State 3 FO | -.191 | -.814 | .420 |  |  |  |  |
| **Step 3** |  |  |  | 1.175 | .073 | .073 | -.003 |
| (Constant) | - | 3.148 | .004 |  |  |  |  |
| State 2 FO | -.248 | -1.398 | .172 |  |  |  |  |
| State 3 FO | -.142 | -.800 | .430 |  |  |  |  |
| **Step 4** |  |  |  | .230 | .053 | .022 | -.020 |
| (Constant) | - | 3.319 | .002 |  |  |  |  |
| State 2 FO | -.230 | -1.316 | .198 |  |  |  |  |
| **Step 5** |  |  |  | - | .000 | .000 | -.053 |
| (Constant) | - | 8.264 | .000 |  |  |  |  |
| Each row represents one step in the hierarchical regression analysis, i.e., after removing a non-significant predictor from the model. FO = fractional occupancy. N = 33. *p < .05, ** p < .01. | | | | | | | |

**Suppl. Table 3 General hallucination proneness as dependent variable and mean duration values of states 1-5 as predictors.**

|  | **Coefficients** | | | **Model summary** | | | |
| --- | --- | --- | --- | --- | --- | --- | --- |
| **Predictor** | **β** | **t** | **p** | **F** | **R^2^** | **Adj. R^2^** | **R^2^**Δ |
| **Step 1** |  |  |  | .771 | .125 | -.037 | .125 |
| (Constant) | **-** | .654 | .519 |  |  |  |  |
| State 1 MD | .058 | .285 | .778 |  |  |  |  |
| State 2 MD | -.311 | -1.478 | .151 |  |  |  |  |
| State 3 MD | .046 | .299 | .821 |  |  |  |  |
| State 4 MD | .279 | 1.414 | .169 |  |  |  |  |
| State 5 MD | .150 | .640 | .527 |  |  |  |  |
| **Step 2** |  |  |  | .984 | .123 | -.002 | -.002 |
| (Constant) | - | .967 | .342 |  |  |  |  |
| State 1 MD | .058 | .288 | .775 |  |  |  |  |
| State 2 MD | .294 | -1.515 | .141 |  |  |  |  |
| State 4 MD | .268 | 1.424 | .165 |  |  |  |  |
| State 5 MD | .131 | .608 | .548 |  |  |  |  |
| **Step 3** |  |  |  | 1.326 | .121 | .030 | -.003 |
| (Constant) | - | 1.067 | .295 |  |  |  |  |
| State 2 MD | -.304 | -1.613 | .118 |  |  |  |  |
| State 4 MD | -278 | 1.530 | .137 |  |  |  |  |
| State 5 MD | .160 | .853 | .401 |  |  |  |  |
| **Step 4** |  |  |  | 1.641 | .099 | .039 | -.022 |
| (Constant) | - | 2.099 | .044 |  |  |  |  |
| State 2 MD | -.249 | -1.413 | .168 |  |  |  |  |
| State 4 MD | .243 | 1.378 | .178 |  |  |  |  |
| **Step 5** |  |  |  | 1.344 | .042 | .011 | -.057 |
| (Constant) | - | 2.411 | .022 |  |  |  |  |
| State 2 MD | -.204 | -1.159 | .255 |  |  |  |  |
| **Step 6** |  |  |  | - | .000 | .000 | -.042 |
| (Constant) | - | 8.264 | .000 |  |  |  |  |
| Each row represents one step in the hierarchical regression analysis, i.e., after removing a non-significant predictor from the model. MD = mean duration. N = 33. *p < .05, ** p < .01. | | | | | | | |

**Suppl. Table 4 Auditory hallucination proneness as dependent variable and fractional occupancy values of states 1-5 as predictors.**

|  | **Coefficients** | | | **Model summary** | | | |
| --- | --- | --- | --- | --- | --- | --- | --- |
| **Predictor** | **β** | **t** | **p** | **F** | **R^2^** | **Adj. R^2^** | **R^2^**Δ |
| **Step 1** |  |  |  | 1.941 | .217 | -.105 | .217 |
| (Constant) | - | .513 | .612 |  |  |  |  |
| State 1 FO | .391 | -.086 | .704 |  |  |  |  |
| State 2 FO | -.086 | -.384 | .704 |  |  |  |  |
| State 3 FO | -.055 | -.252 | .803 |  |  |  |  |
| State 5 FO | -.085 | -.466 | .645 |  |  |  |  |
| **Step 2** |  |  |  | 2.652 | .215 | .134 | -.002 |
| (Constant) | - | .510 | .614 |  |  |  |  |
| State 1 FO | .431 | 2.253* | .032* |  |  |  |  |
| State 2 FO | -.061 | -.309 | .760 |  |  |  |  |
| State 5 FO | -.087 | -.488 | .629 |  |  |  |  |
| **Step 3** |  |  |  | 4.053 | .213 | .160 | -.003 |
| (Constant) | - | .438 | .664 |  |  |  |  |
| State 1 FO | .461 | 2.827** | .008** |  |  |  |  |
| State 5 FO | -.107 | -.656 | .517 |  |  |  |  |
| **Step 4** |  |  |  | 7.818 | .201 | .176 | -.011 |
| (Constant) | - | -.066 | .948 |  |  |  |  |
| State 1 FO | .499 | 2.796** | .009** |  |  |  |  |
| Each row represents one step in the hierarchical regression analysis, i.e., after removing a non-significant predictor from the model. FO = fractional occupancy. N = 33. *p < .05, ** p < .01. | | | | | | | |

**Suppl. Table 5 Auditory hallucination proneness as dependent variable and mean duration values of states 1-5 as predictors.**

|  | **Coefficients** | | | **Model summary** | | | |
| --- | --- | --- | --- | --- | --- | --- | --- |
| **Predictor** | **β** | **t** | **p** | **F** | **R^2^** | **Adj. R^2^** | **R^2^**Δ |
| **Step 1** |  |  |  | 1.502 | .218 | .078 | .218 |
| (Constant) | - | .627 | .536 |  |  |  |  |
| State 1 MD | .401 | 2.079* | .047* |  |  |  |  |
| State 2 MD | -.199 | -1.003 | .325 |  |  |  |  |
| State 3 MD | -.087 | -.454 | .654 |  |  |  |  |
| State 4 MD | .020 | .110 | .914 |  |  |  |  |
| State 5 MD | -.033 | -.150 | .882 |  |  |  |  |
| **Step 2** |  |  |  | 1.943 | .217 | .105 | .000 |
| (Constant) | - | .718 | .479 |  |  |  |  |
| State 1 MD | .405 | 2.173* | .038* |  |  |  |  |
| State 2 MD | -.192 | -1.043 | .306 |  |  |  |  |
| State 3 MD | -.092 | -.502 | .619 |  |  |  |  |
| State 5 MD | -.042 | -.204 | .840 |  |  |  |  |
| **Step 3** |  |  |  | 2.666 | .216 | .135 | -.001 |
| (Constant) | - | .723 | .476 |  |  |  |  |
| State 1 MD | .389 | 2.321* | .028* |  |  |  |  |
| State 2 MD | -.207 | -1.234 | .227 |  |  |  |  |
| State 3 MD | -.080 | -.470 | .642 |  |  |  |  |
| **Step 4** |  |  |  | 3.991 | .210 | .158 | -.006 |
| (Constant) | - | .578 | .568 |  |  |  |  |
| State 1 MD | .405 | 2.497* | .018* |  |  |  |  |
| State 2 MD | -.222 | -1.367 | .182 |  |  |  |  |
| **Step 5** |  |  |  | 5.949 | .161 | .134 | -.049 |
| (Constant) | - | -.920 | .365 |  |  |  |  |
| State 1 MD | .401 | 2.439* | .021* |  |  |  |  |
| Each row represents one step in the hierarchical regression analysis, i.e., after removing a non-significant predictor from the model. MD = mean duration. N = 33. *p < .05, ** p < .01. | | | | | | | |

**Suppl. Table 6 Auditory verbal hallucination proneness as dependent variable and fractional occupancy values of states 1-5 as predictors.**

|  | **Coefficients** | | | **Model summary** | | | |
| --- | --- | --- | --- | --- | --- | --- | --- |
| **Predictor** | **β** | **t** | **p** | **F** | **R^2^** | **Adj. R^2^** | **R^2^**Δ |
| **Step 1** |  |  |  | 1.616 | .188 | .072 | .188 |
| (Constant) | - | -.156 | .878 |  |  |  |  |
| State 1 FO | .470 | 1.841 | .076 |  |  |  |  |
| State 2 FO | .152 | .665 | .511 |  |  |  |  |
| State 3 FO | .001 | .006 | .995 |  |  |  |  |
| State 5 FO | -.228 | -1.233 | .288 |  |  |  |  |
| **Step 2** |  |  |  | 2.232 | .188 | .104 | .000 |
| (Constant) | - | -.242 | .811 |  |  |  |  |
| State 1 FO | .469 | 2.406* | .023* |  |  |  |  |
| State 2 FO | .152 | .754 | .457 |  |  |  |  |
| State 5 FO | -.228 | -1.257 | .219 |  |  |  |  |
| **Step 3** |  |  |  | 3.108 | .172 | .116 | -.016 |
| (Constant) | - | .563 | .578 |  |  |  |  |
| State 1 FO | .395 | 2.361* | .025* |  |  |  |  |
| State 5 FO | -.177 | -1.061 | .297 |  |  |  |  |
| **Step 4** |  |  |  | 5.070 | .141 | .113 | -.031 |
| (Constant) | - | -.318 | .753 |  |  |  |  |
| State 1 FO | .375 | 2.52* | .032* |  |  |  |  |
| Each row represents one step in the hierarchical regression analysis, i.e., after removing a non-significant predictor from the model. FO = fractional occupancy. N = 33. *p < .05, ** p < .01. | | | | | | | |

**Suppl. Table 7 Auditory verbal hallucination proneness as dependent variable and mean duration values of states 1-5 as predictors.**

|  | **Coefficients** | | | **Model summary** | | | |
| --- | --- | --- | --- | --- | --- | --- | --- |
| **Predictor** | **β** | **t** | **p** | **F** | **R^2^** | **Adj. R^2^** | **R^2^**Δ |
| **Step 1** |  |  |  | 1.041 | .162 | .006 | .162 |
| (Constant) | - | .161 | .874 |  |  |  |  |
| State 1 MD | .426 | 2.132* | .042* |  |  |  |  |
| State 2 MD | .010 | .049 | .962 |  |  |  |  |
| State 3 MD | -.090 | -.457 | .651 |  |  |  |  |
| State 4 MD | -.034 | -.176 | .861 |  |  |  |  |
| State 5 MD | -.145 | -.631 | .534 |  |  |  |  |
| **Step 2** |  |  |  | 1.349 | .162 | .042 | .000 |
| (Constant) | - | 1.74 | .863 |  |  |  |  |
| State 1 MD | .424 | 2.191* | 0.037* |  |  |  |  |
| State 3 MD | -.087 | -.477 | .637 |  |  |  |  |
| State 4 MD | -.031 | -.173 | .864 |  |  |  |  |
| State 5 MD | -.140 | -.695 | .493 |  |  |  |  |
| **Step 3** |  |  |  | 1.850 | .161 | .074 | -.001 |
| (Constant) | - | .114 | .919 |  |  |  |  |
| State 1 MD | .419 | 2.225* | .034* |  |  |  |  |
| State 3 MD | -.083 | -.465 | .645 |  |  |  |  |
| State 5 MD | -.132 | -.685 | .499 |  |  |  |  |
| **Step 4** |  |  |  | 2.739 | .154 | .098 | -.006 |
| (Constant) | - | -.281 | .780 |  |  |  |  |
| State 1 MD | .427 | 2.304* | .028* |  |  |  |  |
| State 5 MD | -.112 | -.604 | .550 |  |  |  |  |
| **Step 5** |  |  |  | 5.219 | .144 | .116 | -.010 |
| (Constant) | - | -1.178 | .248 |  |  |  |  |
| State 1 MD | .380 | 2.285* | .029* |  |  |  |  |
| Each row represents one step in the hierarchical regression analysis, i.e., after removing a non-significant predictor from the model. MD = mean duration. N = 33. *p < .05, ** p < .01. | | | | | | | |


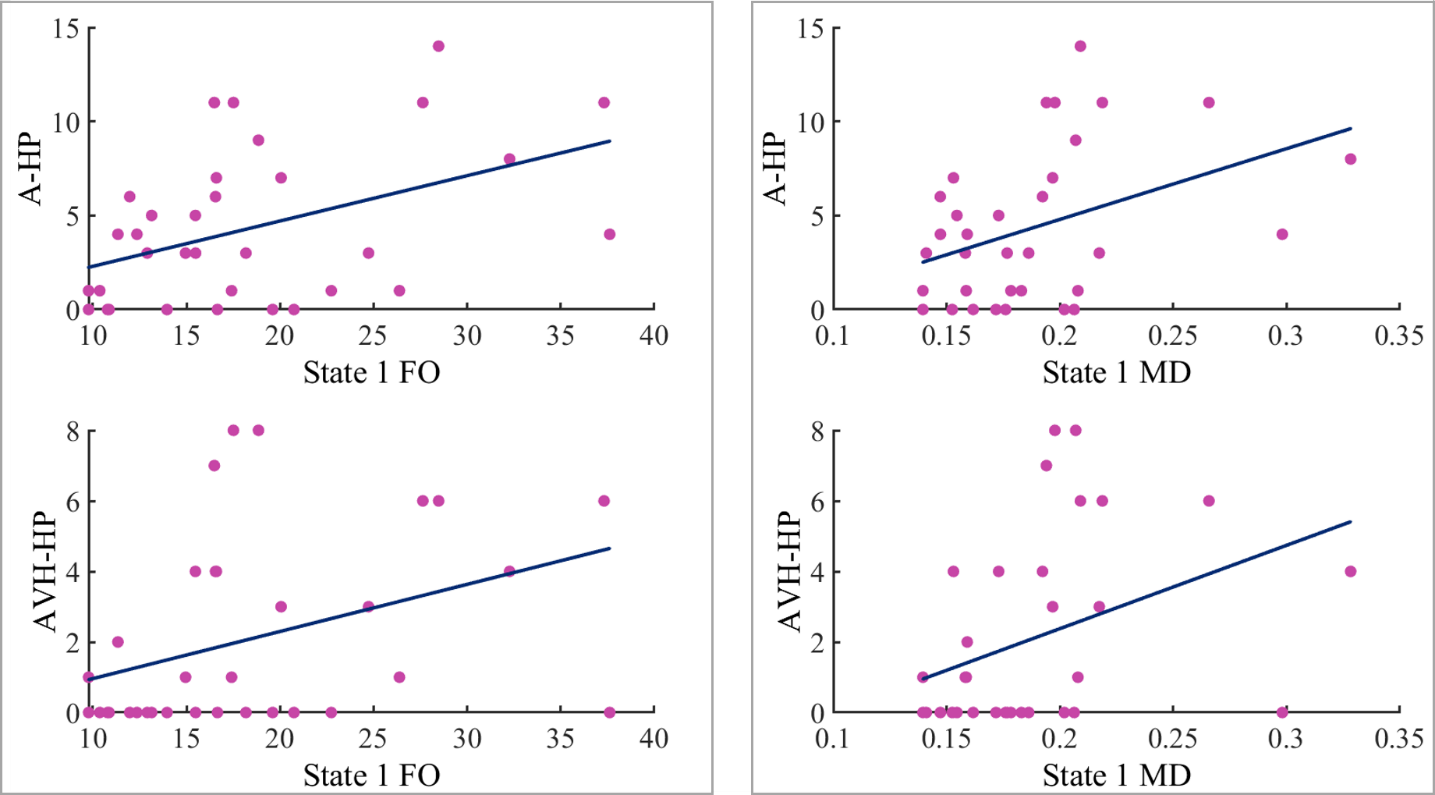


**Suppl. Fig. 3 Scatterplots with fitted regression line of significant relationships of state 1 dynamics and hallucination proneness scores**

Auditory-verbal hallucination proneness (A-HP); Auditory-verbal hallucination proneness (AV-HP); Fractional occupancy (FO); Mean duration (MD).

1. **Additional results of the source reconstruction**

**Suppl. Table 8 Active sources after thresholding (highest 25 % of activation) per state**

| **State 1** | **State 2** | **State 3** | **State 4** | **State 5** |
| --- | --- | --- | --- | --- |
| Frontal Inf Oper L  Rolandic Oper L  Rolandic Oper R  Cingulum Mid L  Cingulum Post L  Cingulum Post R  Calcarine R  Cuneus L  Cuneus R  Occipital Sup R  Occipital Mid R  Postcentral L  Postcentral R  Parietal Sup L  Parietal Sup R  Parietal Inf L  Parietal Inf R  SupraMarginal L  SupraMarginal R  Angular L  Angular R  Precuneus L  Precuneus R  Paracentral Lobule L  Paracentral Lobule R  Caudate L  Thalamus L  Heschl L  Heschl R  Temporal Sup L  Temporal Sup R  Temporal Mid L  Temporal Mid R | Rolandic Oper R  Cingulum Mid L  Cingulum Mid R  Cingulum Post L  Cingulum Post R  Hippocampus R  Calcarine R  Cuneus L  Cuneus R  Lingual R  Occipital Sup R  Fusiform R  Parietal Sup L  Parietal Inf L  Parietal Inf R  SupraMarginal L  Angular L  Precuneus L  Precuneus R  Paracentral Lobule L  Paracentral Lobule R  Thalamus L  Thalamus R  Heschl R  Cerebellum 4 5 L | Precentral L  Frontal Inf Oper L  Rolandic Oper L  Rolandic Oper R  Cingulum Mid L  Cingulum Post L  Cingulum Post R  Calcarine L  Calcarine R  Cuneus L  Cuneus R  Lingual L  Lingual R  Occipital Sup R  Postcentral L  Parietal Sup L  Parietal Inf L  SupraMarginal L  Angular L  Precuneus L  Precuneus R  Paracentral Lobule L  Paracentral Lobule R  Caudate L  Pallidum L  Thalamus L  Heschl L  Heschl R  Temporal Sup L  Temporal Mid L  Temporal Mid R  Vermis 1 2  Vermis 3 | Frontal Inf Oper L  Rolandic Oper L  Cingulum Mid L  Cingulum Mid R  Cingulum Post L  Cingulum Post R  Calcarine R  Cuneus L  Cuneus R  Lingual L  Occipital Sup R  Occipital Mid R  Parietal Sup L  Parietal Sup R  Parietal Inf L  Parietal Inf R  SupraMarginal L  Angular L  Angular R  Precuneus L  Precuneus R  Paracentral Lobule L  Paracentral Lobule R  Caudate L  Putamen L  Pallidum L  Thalamus L  Thalamus R  Heschl R  Temporal Mid R | Precentral R  Frontal Inf Oper L  Rolandic Oper L  Rolandic Oper R  Cingulum Mid L  Cingulum Post L  Cingulum Post R  Calcarine R  Cuneus L  Cuneus R  Occipital Sup R  Postcentral L  Parietal Sup L  Parietal Inf L  SupraMarginal L  SupraMarginal R  Angular L  Precuneus L  Precuneus R  Paracentral Lobule L  Paracentral Lobule R  Thalamus L  Heschl L  Heschl R  Temporal Sup L  Temporal Sup R  Temporal Mid R |

R = right hemisphere; L = left hemisphere.


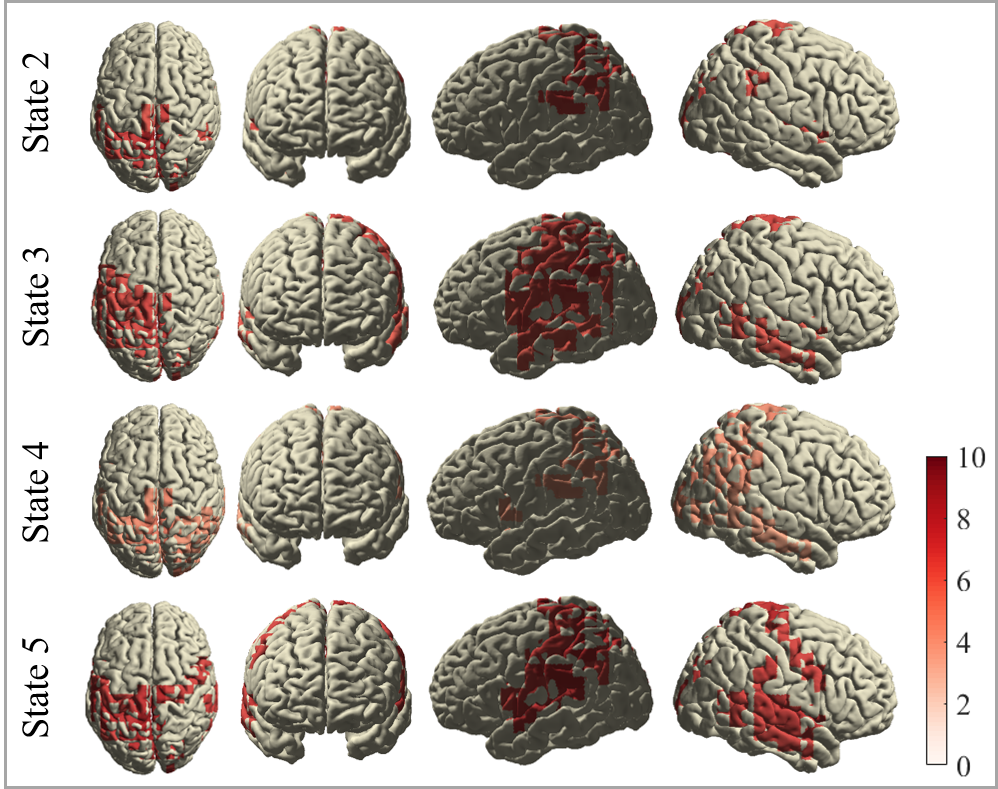


**Suppl. Fig. 4 Cortical distribution of active sources of states 2 - 5**

Activation is expressed as Neural Activity Index (NAI) (Van Veen et al., 1997). Images were generated by i) parcellating the individual source images using the AAL atlas (Tzourio-Mazoyer et al., 2002) into 116 areas, ii) averaging the activity within each parcel across participants, iii) thresholding the mean sources images retaining only the highest 25% of active areas (>/= z-value of 0.675), and iv) interpolating the active sources onto a template mesh provided by Fieldtrip. State 2 and 3 were mostly left-lateralized. State 4 was characterized by source activity in the posterior parietal cortex bilaterally and in the right inferior temporal lobe. State 5 again showed a more asymmetrical activation pattern and was defined by source activity in the left, but not the right, inferior and superior parietal cortex. Additionally, state 5 showed more activation in the right temporal lobes, as compared to the left.


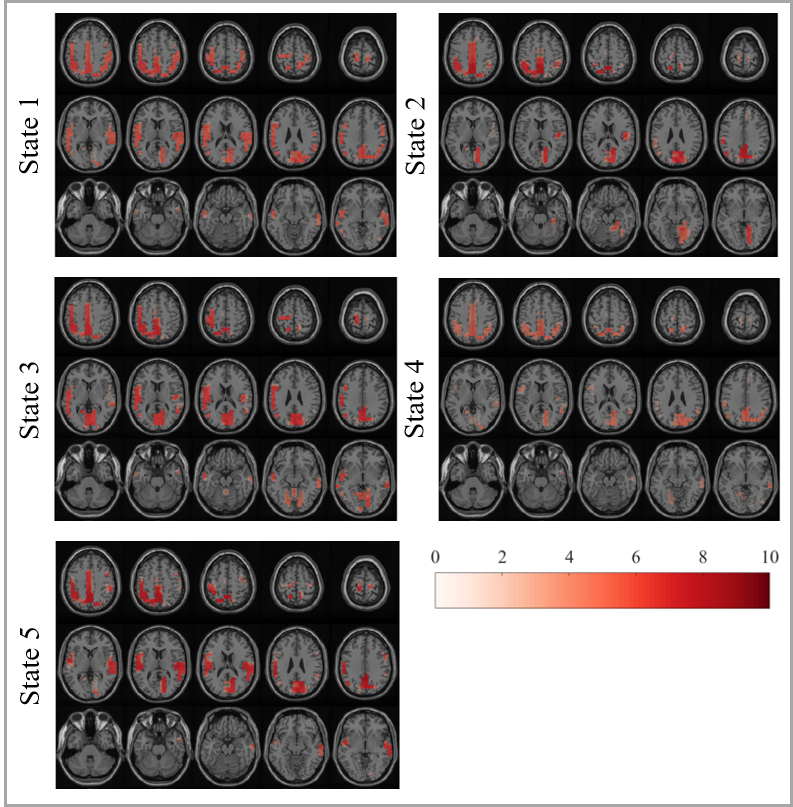


**Suppl. Fig. 5 Axial slices of mean source activation per state**

The cortical slices show the source of highest activation strength per state. Activation is expressed as Neural Activity Index (NAI) (Van Veen et al., 1997). Images were generated by i) parcellating the individual source images using the AAL atlas (Tzourio-Mazoyer et al., 2002) into 116 areas, ii) averaging the activity within each parcel across participants, iii) thresholding the mean sources images retaining only the highest 25% of active areas (>/= z-value of 0.675), and iv) interpolating the active sources onto a template MRI provided by Fieldtrip.

**References**

Chen, G., Lu, G., Xie, Z., & Shang, W. (2020). Anomaly detection in EEG signals: a case study on similarity measure. *Computational intelligence and neuroscience, 2020*.

Collignon, O., Van der Linden, M., & Larøi, F. (2005). Source monitoring for actions in hallucination proneness. *Cognitive neuropsychiatry, 10*(2), 105-123.

de Miras, J. R., Ibáñez-Molina, A., Soriano, M., & Iglesias-Parro, S. (2023). Schizophrenia classification using machine learning on resting state EEG signal. *Biomedical Signal Processing and Control, 79*, 104233.

Drakesmith, M., El-Deredy, W., & Welbourne, S. (2013). Reconstructing coherent networks from electroencephalography and magnetoencephalography with reduced contamination from volume conduction or magnetic field spread. *PloS one, 8*(12), e81553.

Fuchs, M., Kastner, J., Wagner, M., Hawes, S., & Ebersole, J. S. (2002). A standardized boundary element method volume conductor model. *Clinical Neurophysiology, 113*(5), 702-712.

Garrison, J. R., Moseley, P., Alderson-Day, B., Smailes, D., Fernyhough, C., & Simons, J. S. (2017). Testing continuum models of psychosis: No reduction in source monitoring ability in healthy individuals prone to auditory hallucinations. *cortex, 91*, 197-207.

Huggins, C. J., Escudero, J., Parra, M. A., Scally, B., Anghinah, R., De Araújo, A. V. L., . . . Abasolo, D. (2021). Deep learning of resting-state electroencephalogram signals for three-class classification of Alzheimer’s disease, mild cognitive impairment and healthy ageing. *Journal of Neural Engineering, 18*(4), 046087.

Kanemoto, M., Asai, T., Sugimori, E., & Tanno, Y. (2013). External misattribution of internal thoughts and proneness to auditory hallucinations: the effect of emotional valence in the Deese–Roediger–McDermott paradigm. *Frontiers in human neuroscience, 7*, 351.

Larøi, F., & Van Der Linden, M. (2005). Nonclinical Participants' Reports of Hallucinatory Experiences. *Canadian Journal of Behavioural Science/Revue canadienne des sciences du comportement, 37*(1), 33.

Larøi, F., Van der Linden, M., & Marczewski, P. (2004). The effects of emotional salience, cognitive effort and meta‐cognitive beliefs on a reality monitoring task in hallucination‐prone subjects. *British Journal of Clinical Psychology, 43*(3), 221-233.

Launay, G., & Slade, P. (1981). The measurement of hallucinatory predisposition in male and female prisoners. *Personality and Individual Differences, 2*(3), 221-234.

Mao, W., Zhu, J., Li, X., Zhang, X., & Sun, S. (2018). *Resting state eeg based depression recognition research using deep learning method.* Paper presented at the Brain Informatics: International Conference, BI 2018, Arlington, TX, USA, December 7–9, 2018, Proceedings 11.

Murphy, K. P. (2012). *Machine learning: a probabilistic perspective*: MIT press.

Trujillo-Barreto, N. J., Araya, D., Astudillo, A., & El-Deredy, W. (2024). Explicit Modeling of Brain State Duration Using Hidden Semi Markov Models in EEG Data. *IEEE Access*.

Tzourio-Mazoyer, N., Landeau, B., Papathanassiou, D., Crivello, F., Etard, O., Delcroix, N., . . . Joliot, M. (2002). Automated anatomical labeling of activations in SPM using a macroscopic anatomical parcellation of the MNI MRI single-subject brain. *Neuroimage, 15*(1), 273-289.

Van Veen, B. D., Van Drongelen, W., Yuchtman, M., & Suzuki, A. (1997). Localization of brain electrical activity via linearly constrained minimum variance spatial filtering. *IEEE Transactions on Biomedical Engineering, 44*(9), 867-880.

Xu, J., Zheng, Y., Mao, Y., Wang, R., & Zheng, W.-S. (2020). *Anomaly detection on electroencephalography with self-supervised learning.* Paper presented at the 2020 IEEE International Conference on Bioinformatics and Biomedicine (BIBM).
